# Studying the DNA damage response pathway in hematopoietic canine cancer cell lines, a necessary step for finding targets to generate new therapies to treat cancer in dogs

**DOI:** 10.3389/fvets.2023.1227683

**Published:** 2023-08-16

**Authors:** Beatriz Hernández-Suárez, David A. Gillespie, Ewa Dejnaka, Piotr Kupczyk, Bożena Obmińska-Mrukowicz, Aleksandra Pawlak

**Affiliations:** ^1^Department of Pharmacology and Toxicology, Faculty of Veterinary Medicine, Wroclaw University of Environmental and Life Sciences, Wrocław, Poland; ^2^Facultad de Medicina, Instituto de Tecnologías Biomédicas, Universidad de La Laguna, Tenerife, Spain; ^3^Division of General and Experimental Pathology, Department of Clinical and Experimental Pathology, Faculty of Medicine, Wroclaw Medical University, Wroclaw, Poland

**Keywords:** Chk1, Rad51, ATR, Claspin, lymphoma, leukemia

## Abstract

**Background:**

Dogs present a significant opportunity for studies in comparative oncology. However, the study of cancer biology phenomena in canine cells is currently limited by restricted availability of validated antibody reagents and techniques. Here, we provide an initial characterization of the expression and activity of key components of the DNA Damage Response (DDR) in a panel of hematopoietic canine cancer cell lines, with the use of commercially available antibody reagents.

**Materials and methods:**

The techniques used for this validation analysis were western blot, qPCR, and DNA combing assay.

**Results:**

Substantial variations in both the basal expression (ATR, Claspin, Chk1, and Rad51) and agonist-induced activation (p-Chk1) of DDR components were observed in canine cancer cell lines. The expression was stronger in the CLBL-1 (B-cell lymphoma) and CLB70 (B-cell chronic lymphocytic leukemia) cell lines than in the GL-1 (B-cell leukemia) cell line, but the biological significance of these differences requires further investigation. We also validated methodologies for quantifying DNA replication dynamics in hematopoietic canine cancer cell lines, and found that the GL-1 cell line presented a higher replication fork speed than the CLBL-1 cell line, but that both showed a tendency to replication fork asymmetry.

**Conclusion:**

These findings will inform future studies on cancer biology, which will facilitate progress in developing novel anticancer therapies for canine patients. They can also provide new knowledge in human oncology.

## 1. Introduction

Comparative oncology studies cancer across a range of animal species. Thanks to that, it can provide new insights into cancer development and risk factors that may also affect humans. According to the American Veterinary Medical Association, about half of the dogs aged over 10 years will suffer from cancer (1) and in the United States, around 4.2 million dogs are diagnosed with cancer each year (2). With this huge number of patients and a shorter life span than humans, the possibility of completing a clinical trial testing new therapies in canine patients is really promising. Due to biological similarities between cancers in humans and dogs, the results of such trials could potentially be extended to human medicine (3).

Several fundamental regulatory cellular processes are frequently altered in cancer. Disturbances in the functioning of the DNA Damage Response (DDR) pathway are often connected with carcinogenesis (4–11) and resistance to genotoxic stress (12–15), but they also present an opportunity to be used as target for anticancer therapies. Such a therapeutic approach includes the use of DDR inhibitors to overcome cell resistance to genotoxic therapies, or documenting variations in the expression of DDR proteins as potential markers of sensitivity to specific therapies in oncological patients (7, 16–18). Thus, there is a need to validate reagents and molecular techniques for use in canine cells, which will facilitate comparative oncology research.

**Figure 1:**
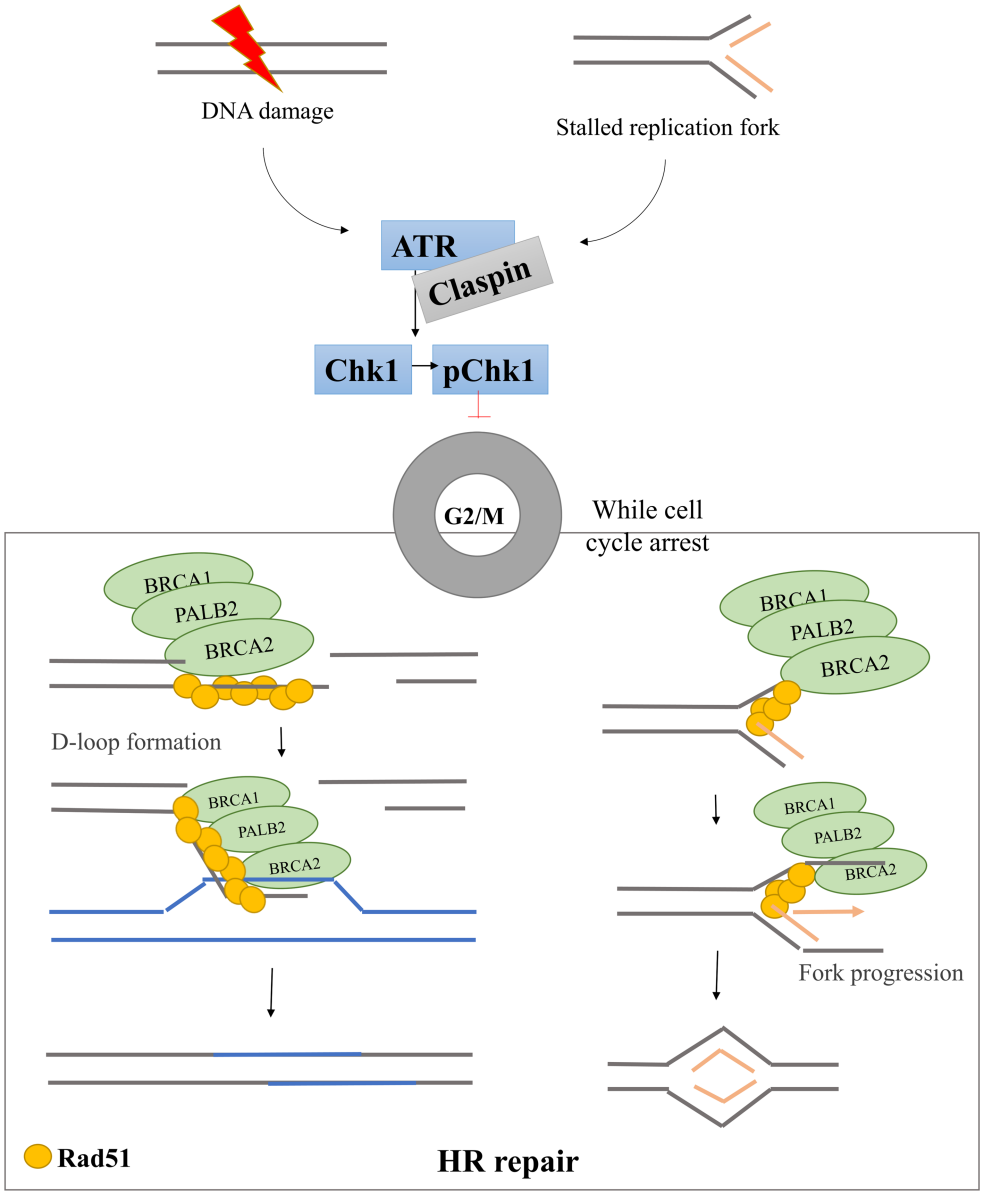
DNA damage response pathway scheme. After DNA damage and/or replication fork stalling, the DDR pathway is activated. ATR is phosphorylated and activates Chk1 through phosphorylation. Activated Chk1 induces cell cycle arrest in G2/M phases allowing the BRCA1-PALB2-BRCA2 triad to recruit Rad51 to the damage site. Rad51 forms filaments that bind DNA and promote D-loop formation allowing the HR repair pathways to start.

The DDR is one of the pathways whose dysfunction can lead to cancer. ATR and Chk1 comprise the principal DDR pathway available to most cancer cells that lack functional p53, which is found altered in almost 50% of human cancers and has also been reported in a variety of canine cancers (19, 20). The ATR-Chk1-Claspin pathway has been found to be upregulated in cancer cells, as compared with non-cancerous cells in humans (6), therefore its inhibition presents an attractive target for new-generation cancer therapies (18, 21). In normal circumstances, the DDR plays a fundamental role in the regulation of cell cycle progression and DNA replication regulation (Figure 1) (22). For example, after DNA damage or during replication stress, thanks to the activation of various DDR components, it is possible to prevent defective cells from dividing by inducing cell cycle arrest. To this end, ataxia telangiectasia mutated and Rad3-related (ATR) kinase phosphorylates and activates checkpoint kinase 1 (Chk1), which induces cell cycle arrest (23). During this complex process, an important mediator protein called Claspin helps to activate Chk1 (13, 24). While the cell cycle arrest continues, the DDR system cooperates to recruit the repair machinery, including proteins involved in homologous recombination (HR), to repair any DNA damage that has occurred. An important component here is the BRCA1-PALB2-BRCA2 complex, which recruits the recombinase Rad51 to form filaments and bind damaged DNA to form a D-loop structure (two strands of a double strand DNA that are separated by a third strand) (25–27). Rad51 responds to replication stress in three ways: (1) by helping promote fork reversal when DNA polymerase progression on a single-stranded DNA (ssDNA) template is blocked (e.g., by DNA breaks), (2) by protecting the ssDNA ends from being degraded by endonucleases, and (3) by promoting restart of replication fork progression (28).

To facilitate research into the significance of DDR pathway disturbances in cancer, as well as to inform studies on the development of new therapies targeting the DDR in dogs, we conducted a series of experiments on canine lymphoma/leukemia cell lines to assess (1): the expression of transcripts of DDR components by RNA sequencing in two selected canine cancer cell lines (2), the basal expression levels of key proteins involved in the DDR (ATR, Claspin, Chk1, Rad51), together with checking the feasibility of using commercially available antibodies, and (3) the functionality of the DDR pathway in canine model cells by assessing the DDR pathway activation after DNA damage, using the DNA damaging agent etoposide (detection of γH2AX and p-Chk1). Finally, we performed DNA combing assays to assess DNA replication dynamics in canine lymphoma/leukemia cells by directly visualizing replication fork progression rates and replication origin firing.

## 2. Materials and methods

### 2.1. Cells and cell culture

A panel of canine lymphoma/leukemia cell lines: CLBL-1 (B-cell lymphoma), CLB70 (B-cell chronic lymphocytic leukemia), and GL-1 (B-cell leukemia) was used in this study. The CLBL-1 cell line was a gift from Barbara Rütgen from the Institute of Immunology, Department of Pathobiology from the University of Vienna (29), the GL-1 cell line was received from Yasuhito Fujino and Hajime Tsujimoto from the Department of Veterinary Internal Medicine at the University of Tokyo (30), and the CLB70 cell line (31) was established with the participation of researchers from our laboratory; the studies involving animals participants were reviewed and approved by the New York Academy of Sciences *Ad Hoc* Committee on Animal Research and were approved by the First Local Committee for Experiments with the Use of Laboratory Animals, Wroclaw, Poland (approval no. 24/2014).

The culture medium RPMI 1640 (Institute of Immunology and Experimental Therapy, Polish Academy of Science, Wrocław, Poland) was used for the CLBL-1 and GL-1 cell lines, and Advanced RPMI (Gibco, Grand Island, NY, United States) for the CLB70 cell line. The culture media were supplemented with 2 mM L-glutamine (Sigma Aldrich, Steinheim, Germany), 100 U/mL of penicillin, 100 μg/mL of streptomycin (Sigma Aldrich, Steinheim, Germany), and 10–20% heat-inactivated fetal bovine serum (FBS; Gibco, Grand Island, NY, United States). The cells were cultured in an atmosphere of 5% CO_2_ and 95% humidified air, at 37°C in 25 cm^2^ cell culture flasks (Corning, New York).

### 2.2. RNA sequencing

RNA was obtained from cultures of the selected cell lines CLBL-1 and GL-1 during unperturbed growth and sequenced by Novogene (United Kingdom). The expected number of Fragments Per Kilobase of transcript sequence per Millions of base pairs sequenced (FPKM) was used to calculate relative gene expression (32). The DDR related GO lists used for the intersection analysis were those presented in Table 1, the gene set information was obtained from the GSEA database (33, 34).

**Table 1:** DDR related GO lists.

Gene set	Species
AMUNDSON_DNA_DAMAGE_RESPONSE_TP53	Human
GOBP_DNA_DAMAGE_RESPONSE_SIGNAL_TRANSDUCTION_BY_P53_CLASS_MEDIATOR	Human
GOBP_DNA_DAMAGE_RESPONSE_SIGNAL_TRANSDUCTION_BY_P53_CLASS_MEDIATOR	Mouse
GOBP_DNA_DAMAGE_RESPONSE_SIGNAL_TRANSDUCTION_BY_P53_CLASS_MEDIATOR_RESULTING_IN_CELL_CYCLE_ARREST	Human
GOBP_DNA_DAMAGE_RESPONSE_SIGNAL_TRANSDUCTION_BY_P53_CLASS_MEDIATOR_RESULTING_IN_CELL_CYCLE_ARREST	Mouse
GOBP_DNA_DAMAGE_RESPONSE_SIGNAL_TRANSDUCTION_RESULTING_IN_TRANSCRIPTION	Human
GOBP_DNA_DAMAGE_RESPONSE_SIGNAL_TRANSDUCTION_RESULTING_IN_TRANSCRIPTION	Mouse
GOBP_NEGATIVE_REGULATION_OF_DNA_DAMAGE_RESPONSE_SIGNAL_TRANSDUCTION_BY_P53_CLASS_MEDIATOR	Human
GOBP_NEGATIVE_REGULATION_OF_DNA_DAMAGE_RESPONSE_SIGNAL_TRANSDUCTION_BY_P53_CLASS_MEDIATOR	Mouse
GOBP_POSITIVE_REGULATION_OF_DNA_DAMAGE_RESPONSE_SIGNAL_TRANSDUCTION_BY_P53_CLASS_MEDIATOR	Human
GOBP_POSITIVE_REGULATION_OF_DNA_DAMAGE_RESPONSE_SIGNAL_TRANSDUCTION_BY_P53_CLASS_MEDIATOR	Mouse
GOBP_POSITIVE_REGULATION_OF_DNA_DAMAGE_RESPONSE_SIGNAL_TRANSDUCTION_BY_P53_CLASS_MEDIATOR_RESULTING_IN_TRANSCRIPTION_OF_P21_CLASS_MEDIATOR	Human
GOBP_REGULATION_OF_DNA_DAMAGE_RESPONSE_SIGNAL_TRANSDUCTION_BY_P53_CLASS_MEDIATOR	Human
GOBP_REGULATION_OF_DNA_DAMAGE_RESPONSE_SIGNAL_TRANSDUCTION_BY_P53_CLASS_MEDIATOR	Mouse
GOBP_REGULATION_OF_DNA_DAMAGE_RESPONSE_SIGNAL_TRANSDUCTION_BY_P53_CLASS_MEDIATOR_RESULTING_IN_TRANSCRIPTION_OF_P21_CLASS_MEDIATOR	Human
GOBP_REGULATION_OF_DNA_DAMAGE_RESPONSE_SIGNAL_TRANSDUCTION_BY_P53_CLASS_MEDIATOR_RESULTING_IN_TRANSCRIPTION_OF_P21_CLASS_MEDIATOR	Mouse
REACTOME_P53_DEPENDENT_G1_DNA_DAMAGE_RESPONSE	Human
REACTOME_SUMOYLATION_OF_DNA_DAMAGE_RESPONSE_AND_REPAIR_PROTEINS	Human
WP_DNA_DAMAGE_RESPONSE	Human
WP_DNA_DAMAGE_RESPONSE_ONLY_ATM_DEPENDENT	Human
WP_MIRNA_REGULATION_OF_DNA_DAMAGE_RESPONSE	Human
WP_MIRNAS_INVOLVED_IN_DNA_DAMAGE_RESPONSE	Human

### 2.3. Treatments

DNA damage was induced by treatment with etoposide (Sigma Aldrich, United States) (a topoisomerase II inhibitor), at 20 μM for 2 h. Treatment conditions were selected based on literature (35) and previous preliminary (Supplementary Figure S5) analysis.

### 2.4. Western blot

3 x 10^5^cells/mL were cultured in 10 mL of media in a 25 cm^2^ culture flask per condition. After 48 h of incubation, the samples were lysed in urea/ SDS buffer (composed of 900 μL of 7 M urea, 25 μL of 5 M NaCl, 25 μL 2 M Tris–HCl (pH = 8), 50 μL 20% SDS), and run in 8–12% bis-tris acrylamide gels prepared using a BioRad Mini-PROTEAN Tetra Vertical Electrophoresis Cell system. The samples were transferred to nitrocellulose membrane using BioRad Mini Trans-Blot® Cell for wet transfer and BioRad Trans-Blot® Turbo™ Transfer System device for semi-dry transfer.

The antibodies used in the study were selected based either on available literature data on reactivity with canine cells (Table 2) or comparison of protein sequence homology, and preliminary test results involving a comparison of the observed bands with the predicted molecular mass (kDa) of the protein of interest. Goat Anti-Mouse Immunoglobulins/HRP (#P0447 at 1:20000 concentration in TBS-T solution) and Goat Anti-Rabbit Immunoglobulins/HRP (#P0448 at 1:10000 concentration in TBS-T solution) were used as secondary antibodies. Both secondary antibodies were from Dako, now part of Agilent (United States, Santa Clara).

**Table 2:** Antibody list showing percentage protein identity between human and dog DDR components.

Protein	Clone	Ref. catalog	Dilution used in the study	% homology*		Literature
				Total protein	Epitope region	
Chk1	G-4	sc-8,408	1:1000 in 3% BSA in TBS-T	96.2		(36–39)
Phospho-Chk1 (SER345)	133D3	#2348	1:1000 in 3% BSA in TBS-T	96.2	100	(36, 38–41)
β-Actin	C4	sc-47,778	1:1000 in 3% milk in TBS-T	97.22	100	(42–47)
ATR	C-1	sc-515,173	1:800 in 3% BSA in TBS-T	94.75	100	(39, 40, 48)
Rad51	G-9	sc-377,467	1:600 in 3% BSA in TBS-T	99.12	100	(41, 49, 50)
Claspin	B-6	sc-376,773	1:800 in 3% BSA in TBS-T	84.47	88	(40)
Anti-gamma H2AX	9F3	ab26350	1:1000 in 3% BSA in TBS-T	99.17	100	(43, 44, 48, 51, 52)

* Basic Local Alignment Search Tool (BLAST) of protein sequences. Antibodies immunogen sequences were analyzed in BLAST® from National Center for Biotechnology Information (NCBI) (www2) (53).

Values are given for both total protein and, where known, for the specific polypeptide region used as immunogen to generate each antibody.

### 2.5. qPCR

#### 2.5.1. Bioinformatic sequence analysis and primer design

The *Canis lupus familiaris* nucleotide accession number sequences for mRNA of the target genes (TGs): Atr, Claspin, and six housekeeping genes (HKGs): Actb, Ppia, and Rplp0 were taken from the Nucleotide Center for Biotechnology Information (NCBI) database (NCBI, United States). The sequences were transferred into the Universal Probe Library. The designed primers and their amplified sequences were additionally verified for their specificity in the Nucleotide Basic Local Alignment Search Tool - Nucleotide-BLAST (NCBI, USA). Gene names, primer sequences for TGs and HKGs, amplicon size, as well their respective gene accession numbers are summarized in Table 3.

**Table 3:** Gene names, forward (F) and reverse (R) primer sequences, amplicon nucleotide (nt) sizes with their respective gene accession numbers.

Gene name	Forward (F) and Reverse (R) primer sequences	Amplicon size (nt)	Gene accesion number
Target Genes (TGs):			
ATR	F: ACCAGACAGCCTACAATGCT R: CCACTTTGCCCTCTCCACAT	77	XM_038432561.1
CLSPN	F: CGCACAAAGCCAGGTGAAAA R: CGTTCCTCATGCCTACGGAG	80	XM_539598.6
Housekeeping genes (HKGs):			
ACTB	F: CGCAAGGACCTCTATGCCAA R: CTTCTGCATCCTGTCAGCGA	78	NM_001195845.3
PPIA	F: TTTGGCAAGGTCAAGGAGGG R: TGGTCTTGCCATTCCTGGAC	73	XM_038689274.1
RPLP0	F: ACATGCTGAACATCTCCCCC R: CAGGGTTGTAGATGCTGCCA	80	XM_038436104.1

#### 2.5.2. RNA isolation and reverse transcription

A total of 1×10^7^ cells cultured in 10 mL from the CLBL-1, CLB70, and GL-1 lymphoma cell lines were centrifuged at 300 g, 4° C, and resuspended in 500 μL of TRIzol reagent (Invitrogen, United States). The cells were immediately transferred to a low-temperature freezer and stored in Eppendorf tubes at −80° C for further analysis. Total RNA isolation was performed using Total RNA Zol-Out™ D (A&A Biotechnology, Poland) according to the protocol provided in the isolation kit. Briefly, the cells were removed from the low-temperature freezer and thawed on ice for 30 min. After that, 167 μL of ultra pure molecular biology water (A&A Biotechnology, Poland) were added, and the sample was mixed by inversion. Next, the cells were spun for 10 min at 10000 rpm. The supernatant was mixed with 1 volume of 96–100% ethanol (Stanlab, Poland) and gently agitated until a homogenous solution was obtained. The supernatants from each tube were transferred into new tubes with an RNA membrane binding column and were centrifuged through the column for 1 min at 10000 rpm and 4° C. The columns were rinsed with 700 μL washing A2W buffer for 2 min at 10000 rpm. DNA digestion for 15 min at 37° C in a thermoblock was performed using DNAse according to the manufacturer’s protocol. The enzymatic activity of the digestive buffer was inhibited by adding 700 μL of R81 buffer and centrifugation [1 min at 10000 rpm at room temperature (RT)]. The filtrate was collected and loaded again onto the column. The membranes were rinsed twice with 700 μL and 200 μL of A2W buffer, centrifuged as described above, and transferred into new Eppendorf tubes. Then, 40 μL of sterile water were added, and after 3-min incubation at RT the tubes with the membranes were centrifuged as above. RNA quality and quantity were estimated using Implen NanoPhotometer (Eppendorf, Germany), and only the samples with a 260/280 nm absorbance coefficient between 1.8 and 2.1 were used for the final experiments. The TranScriba noGenome Kit (A&A Biotechnology, Poland) was used to perform reverse transcription, according to the manufacturer’s recommendations in the MJ Research PTC-100 thermocycler (Marshall Scientific, United States). First, 1 μg of total RNA was mixed with the noGenome master mix. After a 10-min incubation at 42° C, 7 μL of the mentioned mix were added to the RT master mix. The RT master mix included 4 μL of TranScriba buffer, 0.5 μL of RNAse inhibitor, 2 μL of dNTP, 1 μL of starter oligo (dT), 4 μL of TranScriba reverse transcriptase and 1.5 μL of sterile water for one reaction. The reverse transcription protocol was as follows: the first step of 60 min at 42°C, the second step of 5 min at 70°C, and the final step of 5 min at 4°C. The obtained cDNA was stored at −20°C.

#### 2.5.3. Gene expression analysis using real-time PCR

The real-time PCR gene expression analyzes were performed in triplicate from three independent cell cultures. The reaction mix (per well) included 5 μL of RT PCR Mix SYBR® (A&A Biotechnology, Poland), 0.5 μM of forward and reverse primers (Eurofins Genomics AT GmbH, Poland), and 1 μL of cDNA diluted with molecular biology water (16.65 ng cDNA per well). Real-time PCR was performed using the LightCycler 480 II (Roche Molecular Systems Inc., United States) instrument under the following conditions: pre-incubation at 95°C for 10 min, 50 cycles of amplification: 10 s at 95°C for denaturation, 30 s at 60°C for annealing, and 15 s at 72°C for elongation. The gene detection analyzes and primer specificity were further improved by melting curve analysis. The gene expression was categorized using the following scale:

“0” lack of gene expression, Ct values above 35.

“1” very low gene expression, Ct values between 30 and 35.

“2” low gene expression, Ct values between 28 and 30.

“3” regular gene expression, Ct values between 22 and 28.

“4” high gene expression, Ct values between 15 and 22.

“5” very high gene expression, Ct values below 15.

### 2.6. DNA combing assay

A total of 1.6×10^6^ cells were cultured in 10 mL of media, then pulse-labeled with 5-iodo-2′-deoxyuridine (IdU) at 25 μM, followed by 5-chloro-2′-deoxyuridine (CldU) at 200 μM, for 15 min each. The cells were recovered by centrifugation at 300 g after each pulse, and the media were refreshed with each analog. Next, the cells were resuspended in cold PBS and warmed to 42°C, using 5×10^5^ cells per agarose plug. The cells were gently mixed with 1% agarose in PBS and divided into the casting mold to generate the plugs. After treatment with proteinase K (in a buffer made of 1% Sarkosyl, 10 mM Tris pH 7.5, 50 mM EDTA) at 50°C overnight, the DNA was stained with YOYO-1 (5 μM in TE solution for 2–5 min) to check the quality of the fibers. After melting the agarose, the extracted DNA was poured into a reservoir, where a coverslip was inserted, and the DNA fibers were stretched. The resulting fibers were visualized by immunofluorescence detecting the IdU and CldU analogs with red and green antibodies. The coverslips were incubated for 45 min with murine anti-BrdU (IdU, ref. 34,780 Becton Dickinson, United States), and rat anti-BrdU (CIdU, Eurobio ref. ABC117-7513, France), as primary antibodies for the analogs, and for 30 min with goat anti-mouse IgG1 Alexa 564 (ref. A21123 Molecular Probes, Thermo Fisher Scientifics, United States) and chicken anti-rat Alexa 488 (ref. A21470 Molecular Probes, Thermo Fisher Scientifics, United States) as secondary antibodies for the analogs. The coverslips were incubated for 30 min with autoanti-ssDNA DSHB by Voss, E.W. (Hybridoma Product autoanti-ssDNA) autoanti-ssDNA DSHB by Voss, E.W. (DSHB, United States) to detect whole DNA fibers, and then for 30 min with a secondary antibody goat anti-mouse IgG2a Alexa 647 (ref. A21241 Molecular Probes, Thermo Fisher Scientifics, United States). Image acquisition was performed with a 40x objective using a confocal microscope (DM6000; Leica). The fork velocity (FV) was calculated by multiplying the length of the green track of the fiber in micrometers by 2 to obtain Kb, and dividing it by 15 min (time of pulse). Fork asymmetry (FA) was calculated by dividing the long track by the short track. For this analysis, only the first analog incorporation tracks (green tracks) were considered.

### 2.7. Statistical analysis

For the combing assay analysis, the Mann–Whitney test was performed to compare the cell lines and analyze potential differences. Scatterplots were prepared to visually represent the differences between the two cell lines.

Statistical analysis was performed using TIBCO Software Inc. (2017) Statistica (data analysis software system), version 13 http://statistica.io.

## 3. Results

### 3.1. RNA-sequencing analysis revealed the presence of principal components of the DDR pathway in canine cell lines

**Figure 2:**
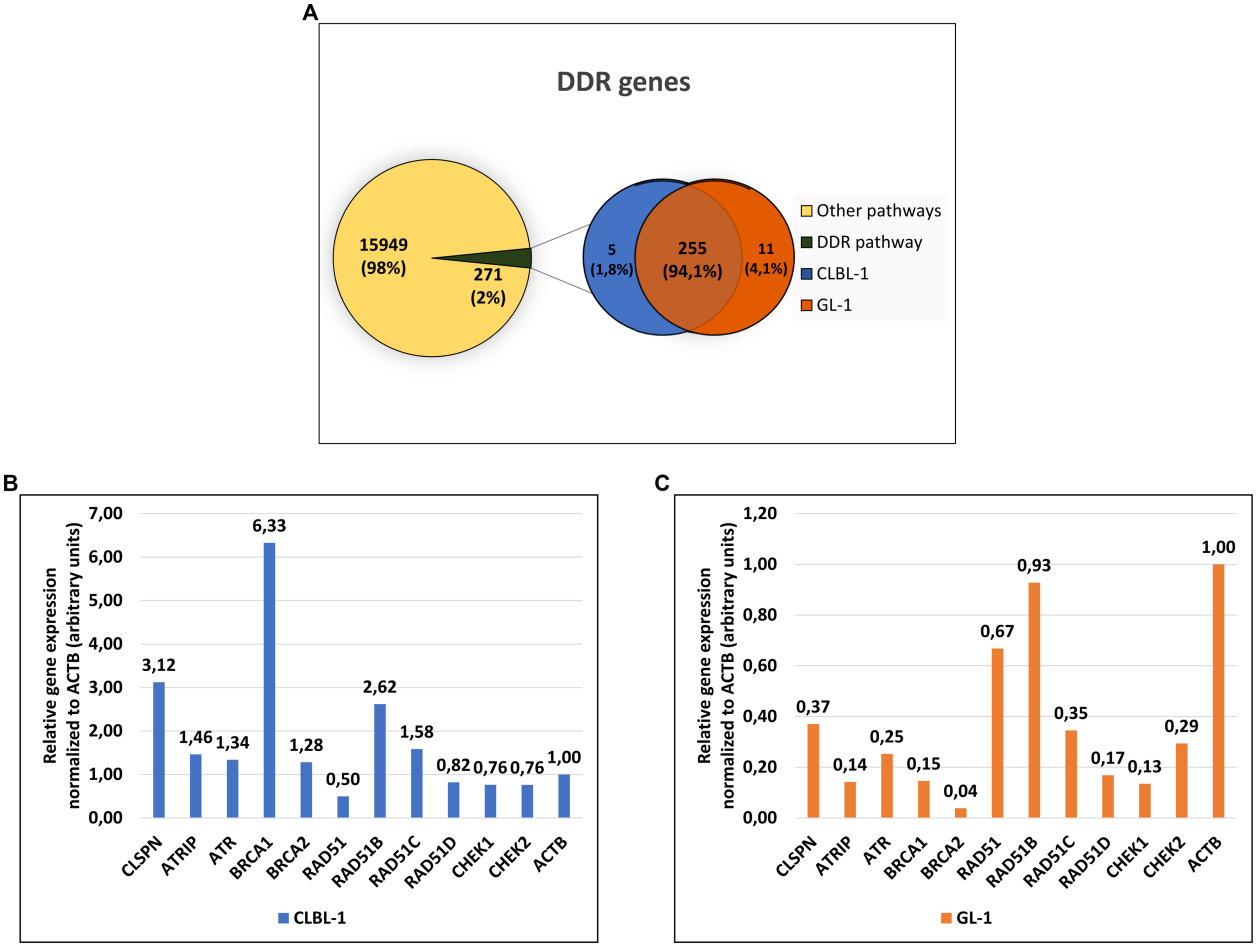
Approximately 2 % of the genes expressed in the lymphoma and leukemia canine cells encode components of the DDR pathway. **(A)** From the 16,220 genes expressed in the CLBL-1 and GL-1 cell lines, only 271 genes are from DDR pathway. **(B,C)** The relative gene expression (calculated by FPKM method) of the ATR-pathway and HR repair components were compared after being normalized to the expression of ACTB gene in CLBL-1 and GL-1, respectively.

The canine lymphoma cell line CLBL-1 and the canine leukemia cell line GL-1 expressed a total of 16,220 genes, from which 271 (~2%) are DDR pathway members. Specifically, the CLBL-1 cell line expressed 260 DDR genes, and the GL-1 cell line 266, with 255 genes in common (Figure 2A). The relative expression of the most important genes with a role in the ATR- and HR-repair pathways was analyzed for both cell lines. Higher expression of all the genes was found in the CLBL-1 than in the GL-1 cell line, except for RAD51 which showed slightly higher expression in the GL-1 cell line (Figure 2B).

### 3.2. Expression and activation of the DDR pathway components in canine cancer cells

**Figure 3:**
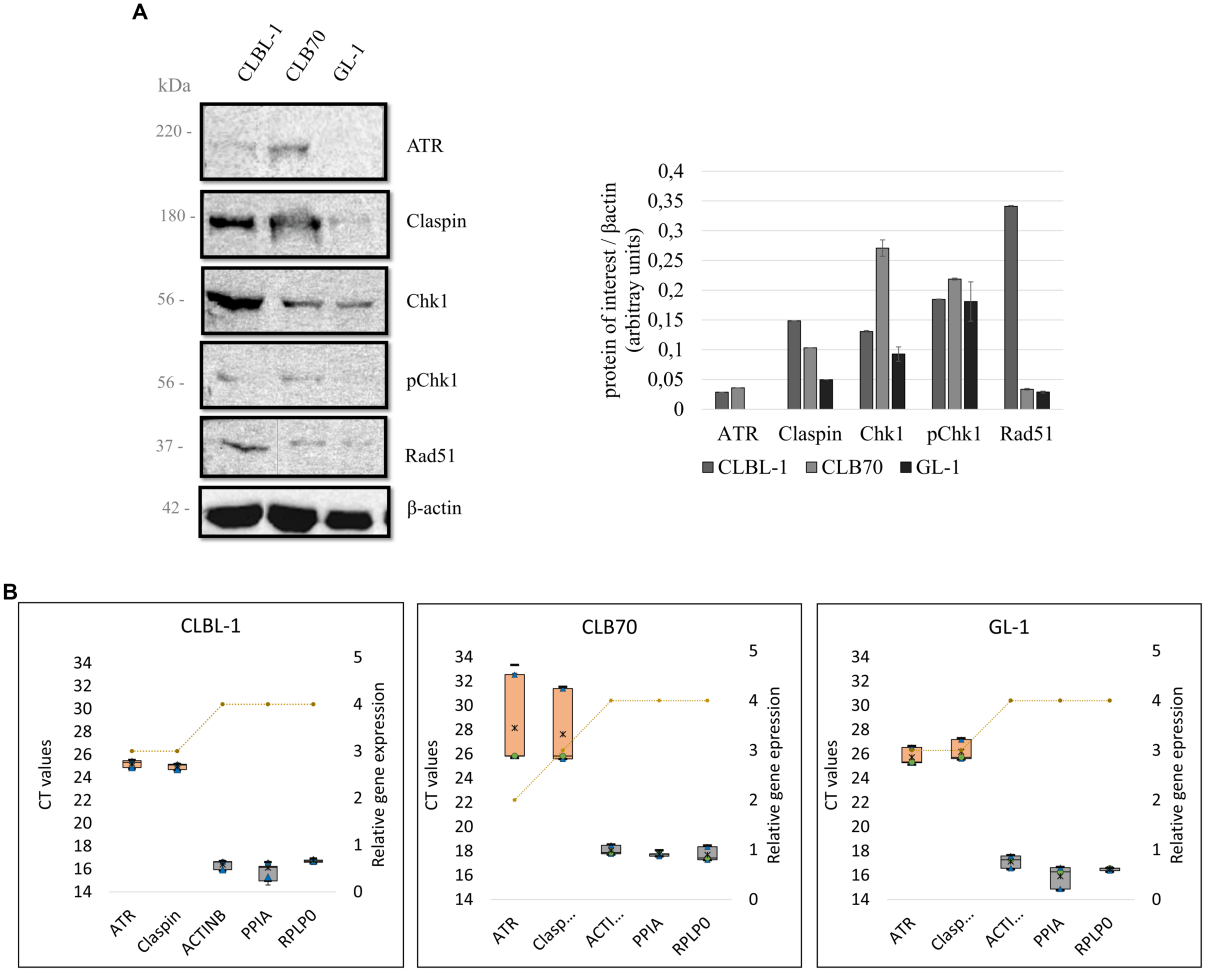
**(A)** Basal expression levels of key DDR proteins in canine lymphoma/leukemia cell lines. Selected proteins of the DDR were analyzed to verify their basal expression level in different canine cell lines. Quantification was performed by normalizing the expression level of the protein of interest to the expression level of the loading control, β-actin. Mean and standard deviations were calculated based on three repetitions from independent experiments. **(B)** Relative gene expression of ATR and Claspin in different cell lines. A box and whiskers plots present raw Ct values obtained with the qPCR for ATR, Claspin, and three selected HKGs: ACTB, PPIA, and RPLP0 (left Y axis). The orange boxes represent the target genes, and the gray ones the HKGs. Minimum and maximum Ct values are represented as black lines, the 25 and 75% quartiles are represented as blue triangles, the median is represented with a green dot, and the mean with a black asterisk. The relative gene expression (right Y axis) of ATR, Claspin and the HKGs for different cell lines is represented by a yellow striped line. The rank for the gene expression is 0 = no expression (Ct above 35), 1 = very low expression (30–35), 2 = low expression (28–30), 3 = regular expression (22–28), 4 = high expression (15–22), and 5 = very high expression (10–15).

Considering that ATR, Claspin, Chk1, and Rad51 are among the most important proteins of the DDR pathway, they are still quite uncharacterized in dogs. Thanks to the progress made in recent years in veterinary medicine, and in particular in veterinary oncology, currently there are some tools available for their study in dogs (for example antibodies and siRNAs).Our initial aim was to identify commercial antibodies against key components (ATR, Claspin, Chk1, Rad51) of the DDR that would be suitable for use in canine cells. Western blot screening was performed to analyze the basal protein expression levels, and to determine whether the pathway activation in response to DNA damage occurs in canine cells (detection of γH2AX and p-Chk1; Figure 3A; Supplementary Figure S4). All the antibodies used in the study were monoclonal antibodies generated using human epitopes as immunogens. The BLAST alignment comparing human and canine protein sequences demonstrated high homology overall and, where known, within the polypeptide region used as immunogen (Table 2; alignments in Supplementary material).

As detecting high-molecular-weight proteins by means of a western blot can be technically challenging (54), a qPCR was performed for the genes encoding ATR and Claspin, in order to obtain more information about the expression of these DDR components in the panel of the analyzed cell lines.

#### 3.2.1. ATR

BLAST alignment demonstrated 94.75% identity between the human and canine ATR protein sequences, which justified the assumption that an antibody directed against a human protein would cross-react with the canine protein. Indeed, the band detected with the antibody ATR C-1 (Santa Cruz sc-515,173) corresponded with the ~220 kDa molecular mass expected for this protein. ATR was readily detected in only two of the three cell lines analyzed, with expression of ATR being higher in CLB70 cells than CLBL-1 and undetectable in GL-1 cells (Figure 3A). As ATR is considered essential for cell proliferation/ survival, and ATR mRNA expression was readily detected in the GL-1 cell line (see below), we assume that the level of ATR protein expression in this cell line is below the limit of detection using this particular method of cell extraction/ WB.

qPCR analysis confirmed that ATR mRNA is expressed in the three cell lines tested, with mean threshold cycle (Ct) values of 25.21 ± 0.27 for the CLBL-1 cell line, 28.15 ± 3.29 for the CLB70 cell line, and 25.72 ± 0.61 for the GL-1 cell line. Although the expression of ATR was substantially lower than the expression of the HKGs (Figure 3B), Ct values below 29 indicate that ATR mRNA is relatively abundant in these cells.

#### 3.2.2. Claspin

In the case of Claspin, BLAST alignment demonstrated 84.47% identity between the human and canine proteins, again supporting the possibility of cross-reactivity of human antibodies with canine proteins. The expression of Claspin was detectable using the Claspin B-6 (Santa Cruz sc-376,773) antibody. The antibody recognized a protein of the expected molecular mass of ~180 kD, thus confirming cross-reactivity with canine Claspin. Claspin expression was observed in all three cell lines, although the expression levels varied. Claspin expression levels were substantially higher in the CLBL-1 and CLB70 cell lines than in the GL-1 cell line, similar to the expression of ATR (Figure 3A).

qPCR analysis showed that the Claspin mRNA was also expressed in the three cell lines, with Ct values of 24.95 ± 0.17 for the CLBL-1 cell line, 27.63 ± 2.70 for the CLB70 cell line, and 26.19 ± 0.74 for the GL-1 cell line. Similar to ATR, the expression of the Claspin mRNA in each case was substantially lower than that of the HKGs (Figure 3B).

#### 3.2.3. Chk1 and p-Chk1

**Figure 4:**
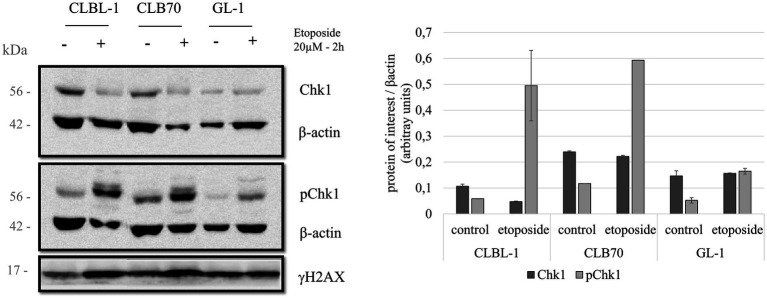
Chk1 protein levels and regulatory phosphorylation after etoposide treatment. Three cell lines were treated with 20 μM of the DNA damaging agent etoposide for 2 h in order to study the changes in the activation of Chk1. Expression levels for total Chk1 and S345-phosphorylated Chk1 were analyzed. Expression levels of γH2AX were also determined as a control for DNA damage. Quantification compares the basal condition against the expression after treatment, normalized to β-actin. Mean and standard deviations were calculated based on three independent replicate experiments.

BLAST alignment for Chk1 protein sequences between humans and dogs showed a high 96.2% identity. This analysis also confirmed that canine Chk1 contains the key regulatory site, serine 345 (S345), that is phosphorylated by ATR to activate Chk1 in response to genotoxic stress. This means that with the use of the tested antibodies Chk1 G-4 (Santa Cruz sc-8,408) and Phospho-Chk1 (SER345) 133D3 (Cell Signaling #2348) directed against human epitopes, both the basal level expression of Chk1 kinase and its activation after DNA damage can be tested in canine cells. Indeed, both antibodies detected proteins of the expected molecular mass (~56 KD) in all three cell lines (Figure 3A). Interestingly, however, the two cell lines with the highest basal expression of Chk1 were again CLBL-1 and CLB70, which also exhibited the highest expression of both ATR and Claspin. Basal levels of active, phospho-S345 Chk1 were also detected in all three cell lines, being highly expressed in CLB70 as compared with the other cell lines (Figure 4). The ability to monitor the level of Chk1 kinase, as well as its activation, will facilitate the development of new molecularly targeted therapies in canine oncology.

#### 3.2.4. Rad51

BLAST alignment comparing Rad51 protein sequences demonstrated 99.12% identity between human and canine protein, indicating a high probability that an antibody directed at the human protein will detect the canine homolog. As expected, using Rad51 G-9 (Santa Cruz sc-377,467) antibody, we detected a band of ~37 kDa corresponding to the expected molecular mass of Rad51. Basal Rad51 recombinase expression was detected in all three cell lines with the highest level of expression in the CLBL-1 cell line (Figure 3A).

### 3.3. Activation of the DDR pathway after etoposide treatment observed as an increase in Chk1 kinase S345 phosphorylation

Next, we analyzed the expression levels and possible regulatory modifications of the canine DDR proteins after inducing activation of the DDR pathway by treating the cells with a classic DNA damaging agent, etoposide. Several studies have reported DDR activation through phosphorylation of Chk1 and an increase of Rad51 expression after treatment with the DNA damaging agent etoposide (55, 56). This information, together with the fact that etoposide is a chemotherapeutic drug used as a treatment for several cancers (57, 58), are the reasons why we decided to select it for our study to induce DNA damage. There were no major changes in the expression levels of total Chk1 after the treatment with etoposide, but the level of Chk1 phosphorylated at S345 varied considerably after exposure to this toxic agent (20 μM for 2 h). The CLBL-1 and CLB70 cell lines, which showed S345 phosphorylation of Chk1 in basal conditions, were also found to present a considerable increase in the phosphorylation levels after the treatment with the DNA damaging agent, while in the GL-1 cell line, this increase was more modest (Figure 4).

### 3.4. DNA combing assay in the canine cells

Advanced techniques, such as DNA combing, can provide much greater insight into DNA replication dynamics by directly visualizing replication fork progression rates and replication origin firing. Due to the important role of ATR-Chk1 in replication, we wished to evaluate the feasibility of performing DNA combing in canine cells.

**Figure 5:**
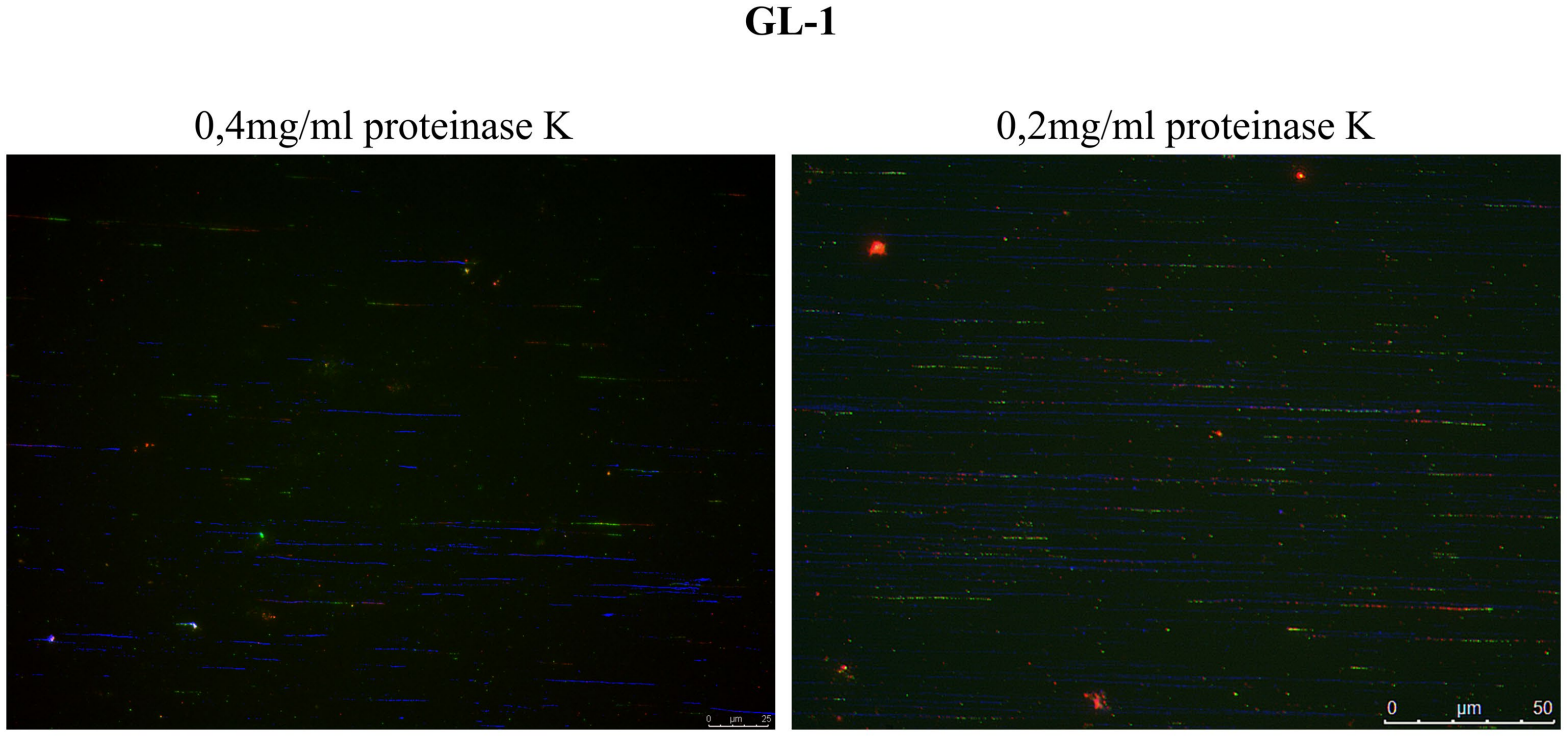
Comparison of fiber integrity after treatment with proteinase K. DNA fibers obtained after treatment with proteinase K at 0.4 mg/mL were frequently broken into relatively short tracts. Lowering the concentration of proteinase K to 0.2 mg/mL improved the quality of the fibers (DNA fibers in blue).

The selected cell lines for this study were GL-1 and CLBL-1, since both present high expression level of Rad51 (Figure 3A; Supplementary Figures S1, S2), which may be related to replication stress. The protocol had to be slightly modified for use with suspension cells. Following the modified protocol, the cells were treated with proteinase K at 0.4 mg/mL. In the first experiment, many fibers were seen to be broken, so subsequently the cells were treated with a lower concentration of proteinase K (0.2 mg/mL), and the quality of the fiber integrity improved (Figure 5).

**Figure 6:**
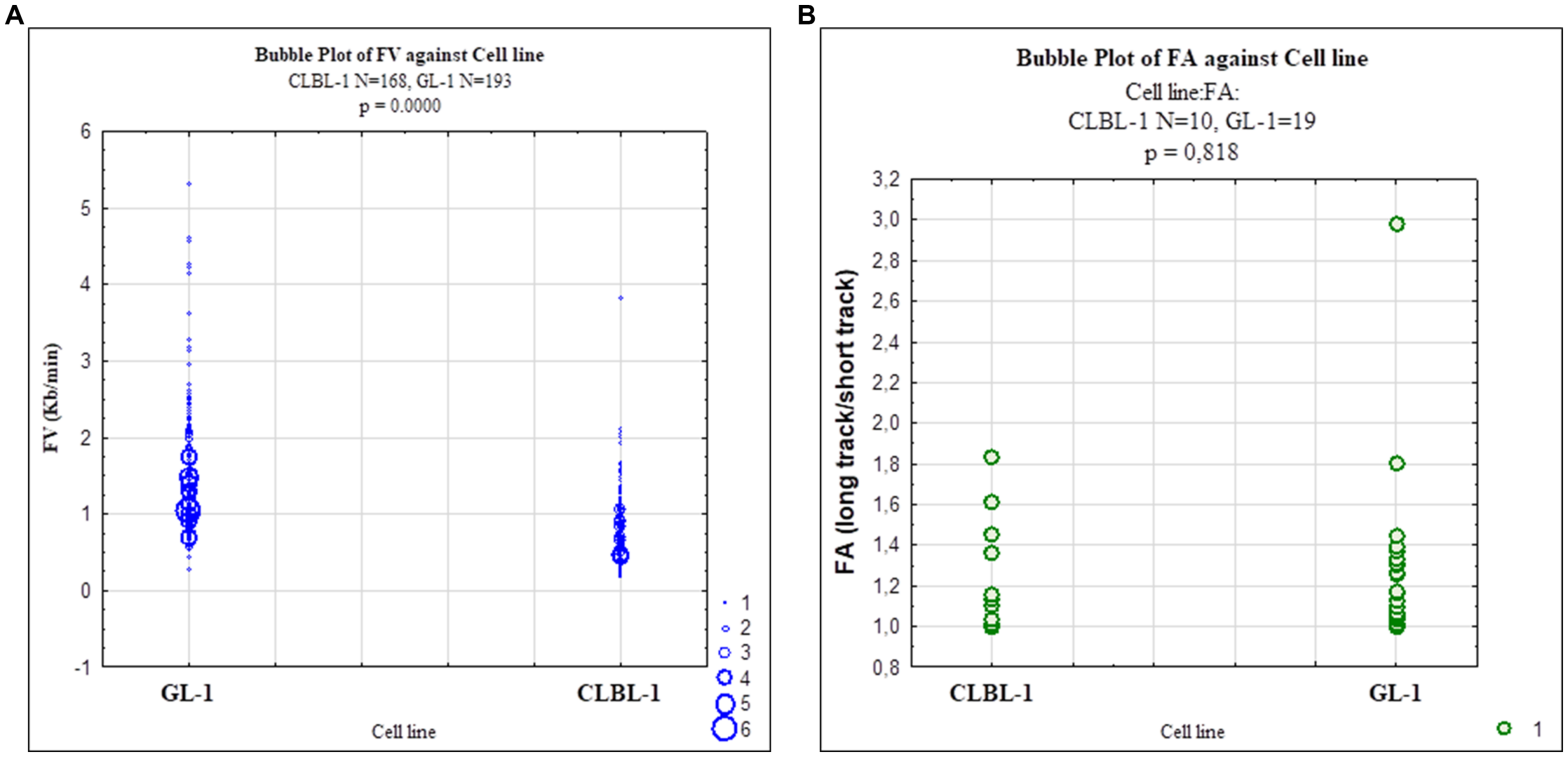
Scatterplots representing replication fork dynamics in CLBL-1 and GL-1 cells The replication fork speed and fork asymmetry were measured in the CLBL-1 and GL-1 cell lines. **(A)** Fork velocity of the GL-1 line was higher than in the CLBL-1 line. **(B)** Fork asymmetry did not differ significantly between the two cell lines.

We then used the DNA combing assay to examine replication dynamics in two selected cell lines, CLBL-1 and GL-1. The data are summarized in Table 4 and Figure 6. The replication fork speed was significantly (*p* = 8.86^−21^) faster in the GL-1 line (1.5 Kb/min) than in the CLBL-1 line (0.86 Kb/min; Figure 6A). When replication fork asymmetry was analyzed, both cell lines had similar means for the calculated ratios, 1.27 for the CLBL-1 and 1.32 for the GL-1 line (Figure 6B), indicating that both exhibited similar levels of replication fork asymmetry.

**Table 4:** Fiber patterns found in the analyzed cells.

Cell line	Progressing fiber	Initiation during the first pulse	Initiation before the first pulse	Termination	Cluster
CLBL-1	128	8	2	24	1
GL-1	124	14	5	21	10

## 4. Discussion

### 4.1. RNA-sequencing analysis revealed the presence of principal components of the DDR pathway in canine cell lines

Due to the important role of the DDR in cancer and the paucity of information about it in canine cancer cells, an RNA-Seq analysis was initially performed in the selected cell lines, CLBL-1 and GL-1, under normal growth conditions. CLBL-1 and GL-1 cell lines were selected for this analysis as representative of common hematopoietic cancers - lymphoma (CLBL-1) and leukemia (GL-1). After sorting the genes by Gene Ontology terms (GO) related to the DDR (Table 1), we found that approximately 2% of expressed genes encode components of the DDR pathway (Figure 2A). Interestingly, the relative expression of most DDR genes in the CLBL-1 line was higher than in the GL-1 cell line, but the expression patterns changed as well. For the CLBL-1 cell line, the most highly expressed genes were *BRCA1*, *CLSPN*, and paralogue B of *RAD51* (*RAD51B*), while *RAD*51 exhibited the lowest relative expression (Figure 2B, in blue). In the case of the GL-1 line, the pattern was different, the highest expression was detected for *RAD51B* and *RAD51*, and the lowest for *BRCA2* (Figure 2C, in orange).

Thus, this RNA-Seq analysis revealed that the canine lymphoma and leukemia cells shared expression of a majority of DDR genes, but that the expression of certain key components differed substantially between the tumor types.

### 4.2. Expression and activation of the DDR pathway components in canine cancer cells

The first screening for the basal expression of DDR proteins (Supplementary Figure S1) indicated significant variations in the protein expression levels for different cancer cell lines. Based on these findings, together with the results of the RNA-Seq analysis (Figure 2), several lymphoma and leukemia cell lines were selected for further experiments. Validation of antibodies that recognize DDR proteins in dogs is needed, and selected commercial antibodies were tested in this study. The BLAST alignment analyzes were performed to check the homology between the human and canine protein sequences. With confirmed high homology, it was assumed that if an antibody detects a single band of correct molecular mass, there is a high probability that this corresponds to the protein of interest, particularly as all the tested antibodies were monoclonal (59, 60).

#### 4.2.1. ATR

ATR was detected in all the cell lines at the mRNA level (Figure 3B). The obtained melting curves showed only one amplicon, which validated the identity of this qPCR product (Supplementary Figure S3). Ct values of 25.21 ± 0.27 for the CLBL-1 cell line, 28.15 ± 3.29 for the CLB70 cell line, and 25.72 ± 0.61 for the GL-1 cell line were observed, indicating robust ATR mRNA expression in all tested cell lines (61).

The BLAST alignment for ATR protein showed a 94.75% protein identity in humans and dogs, meaning that the probability of an antibody designed to recognize the human protein cross-reacting with the dog protein is high. As presented in Figure 3A, the tested ATR antibody recognized the canine protein. To our knowledge, no previous studies on ATR at the protein level have been performed in dogs. Curiously, we found that ATR protein at the basal level was only detected in the CLBL-1 and CLB70 cell lines and not in GL-1, despite clear evidence for ATR mRNA expression in the latter. It is well known however that protein and mRNA levels do not always correlate; several studies demonstrated that the correlation can vary in adenocarcinoma samples (62), can be modulated after treatments with drugs such as rapamycin (63), and may vary during the cell cycle in synchronized cultures (64). What we can conclude from the presented results is that the *ATR* gene is widely expressed in the canine lymphoma/leukemia cell lines. However, as ATR has a high molecular weight of 220 kDa, and high-molecular-weight proteins are difficulted to transfer (54), it is likely that its expression was below the threshold of detection in the GL-1 cell line for unknown reasons. Nuclear extraction and/ or immunoprecipitation are options that could be used to increase the sensitivity of ATR protein detection in GL-1 cells and other canine cancers.

Variations in ATR expression are considered a marker of sensitivity and/or resistance to certain anticancer drugs. High expression of ATR protein has been proposed as a marker of cisplatin sensitivity in patients with bladder cancer (7). Also, in the case of a doxorubicin-resistant canine hemangiosarcoma cell line established to study drug resistance, it was found that the DDR pathway was attenuated, as the mRNAs for ATM, ATR, and Chk1 were significantly decreased, suggesting a possible role for ATR in doxorubicin resistance (15). Another study reported that ATR inhibitors in combination with pyrrolobenzodiazepine (PBD) increased the cytotoxicity of PBD as compared with the drug alone, and helped to overcome the resistance to PBD (65). In other work, human multiple myeloma (MM) cells were treated with MEDI2228, a ligand of the B-cell maturation antigens that induces ATM/ATR-Chk1/2 pathway activation, in combination with different inhibitors of the principal kinases of the DDR (ATM, ATR, and WEE1) (66). The results of that study showed an increase in the toxicity of this ligand when combined with the inhibitor, an interesting example of the use of ATR as a target to induce cell death in MM cells and to abrogate resistance to MEDI2228.

To our knowledge, this is the first time that ATR has been detected in canine lymphoma/leukemia cells providing a new opportunity to study this protein in veterinary oncology.

#### 4.2.2. Claspin

A high percentage of protein identity between the human and canine Claspin proteins was confirmed by BLAST alignment (84.47%). Indeed, Claspin protein expression was detected in all the cell lines of our panel as a single band with a molecular mass of 180 kDa as seen in human cell lines (40). Claspin was highly expressed in the CLBL-1 cells compared to the other cell lines (Figure 3A). Interestingly, the mRNA of the Claspin gene was detected in all three cell lines although at a somewhat lower level in CLB70 cells (Ct value of 27) than in the other lines (Figure 3B). The melting curves showed only one peak, meaning that the primers designed for Claspin specifically amplify a single amplicon (Supplementary Figure S3). Contrary to what we observed in the CLB70 cell line for ATR, Claspin protein expression and its mRNA level in the CLBL-1 cell line were higher than in the other cell lines of the panel. This corresponded with the observation from RNA-Seq that the *CLSPN* gene was among the most highly expressed genes in the CLBL-1 cell line (Figure 2B). This could be an example of a regulated correlation between protein and mRNA expression, which is not as common as one might expect (64).

Claspin has been described to be highly expressed in prostate cancer cells in comparison with non-cancerous prostate cells (6). Many cancer cells present higher expression of the components of the ATR-Claspin-Chk1 pathway, as compared with non-cancerous cells, which can be related to resistance to radiotherapy (13, 14). To our knowledge, only one other study has analyzed Claspin in canine cells (67). In that experiment, a polyclonal antibody included in an apoptosis antibody array kit (Catalog # ARY009) was used. In our study, a monoclonal antibody for Claspin was validated in three different canine cell lines. This indicates the potential utility of this antibody in future veterinary research to test the effects of inhibiting Claspin, or to detect the protein expression level in different tumor samples.

#### 4.2.3. Chk1 and p-Chk1

Chk1 showed a 96.7% identity between the human and canine protein sequences in the BLAST alignment (Supplementary Figure S1). Consistent with this, Chk1 protein was detected in all the cell lines at various levels, with the CLB70 and CLBL-1 lines showing higher expression than the GL-1 line (Figure 3). We also detected high Chk1 mRNA level in the CLBL-1 line (Figure 2B). Interestingly, Chk1 was found to be highly expressed in several tumors, as compared with non-malignant tissues (4, 5). It was described to be overexpressed in human leukemia cells, B-cell lymphomas, and highly expressed in hematopoietic cancers as compared with solid tumors (8, 9, 68), which is consistent with the results obtained in our canine lymphoma/leukemia cell lines. Interestingly, in the CLBL-1 and CLB70 cell lines, the basal level of kinase phosphorylation was much higher than in the GL-1 cell line, suggesting that the response to DNA damage in these two cell lines might be faster and stronger than in the latter.

Upregulation of Chk1 has been proposed as a target for anticancer therapies. Different studies have confirmed Chk1 inhibitors acting as apoptosis inducers in various human and canine tumor cells, indicating, for example, proliferation decrease in human neoplastic B-cells and mast tumor cell (MTC) canine cell lines (68, 69). Currently, there are several Chk1 inhibitors in phase II of clinical trials and the results in human cancers seem promising (8, 70). Knowing that Chk1 is overexpressed in human B-cell lymphomas, and that the antibodies have been validated in our canine cells, we propose the use of canine B-cell lymphoma/leukemia cell lines as a model to study the role of Chk1 in canine B-cell malignancies.

#### 4.2.4. Rad51

The last protein we examined was Rad51. BLAST alignment showed 99.12% Rad51 sequence identity between humans and dogs. This suggests that antibodies designed to recognize human Rad51 will also recognize the canine homolog. In our study, the antibody employed recognized Rad51 protein in all the canine cell lines tested. Its expression was the highest in the CLBL-1 line, both at the protein and gene level (Figures 2B, 3). In the literature, high expression of Rad51 protein is related to genome instability (10, 11), which is a hallmark of cancer. Rad51 is overexpressed in mammary carcinomas, and this is related to metastases in lymph nodes in both humans and dogs (71–74). Rad51 is a protein which has been studied in canine tumors due to its connection with BRCA2, and several studies have documented Rad51 mutations in tumor canine cells (75–77)Bortezomib, a proteasome inhibitor that impairs HR and thus decreases the expression of Rad51, has been used to potentiate the effect of other drugs, such as inhibitors of poly (ADP-ribose) polymerase (iPARP) or MEDI2228. Such combinations can also downregulate Rad51 protein expression, increase cell death, and even help to eradicate tumors in *in vivo* mice models (66, 78). The effects of these drugs on Rad51 function and expression in dogs have not been studied yet. However, as bortezomib is a drug that can be safely administered in dogs (79), treatment with a combination of bortezomib and other DNA damaging agents in canine cell lines could yield useful information to be potentially implemented in the veterinary clinic. Here, a new Rad51 antibody, clone G-9, has been validated in canine cells, and it was also recently validated in other hematopoietic human cell lines (49).

### 4.3. DNA replication dynamics in canine lymphoma/leukemia cell lines

Replication stress arises in cells with DDR defects during the replication of damaged DNA. Replication stress may cause fork asymmetry and consequently, fork stalling and collapse that promotes genetic instability (80). Cancer cells often seem to experience replication stress under conditions where normal cells do not, even when they replicate rapidly (18, 81). A reduction in fork speed has been described under conditions of replication or oxidative stress in cancer cells (82), while pronounced asymmetry of replication forks has been detected in medulloblastoma stem cells (83). Thus, measurement of fork speed and fork asymmetry could help to better understand and describe the phenotype of a cancer cell type, which may later be used in order to choose therapeutic approaches. The ATR-Chk1 pathway is a critical regulator of the replication stress, as its role is to regulate the replication fork progression and stability, presenting potential targets for combination therapies (84) Thus, the analysis of cellular replication in canine cancer cells could bring new opportunities to find targets for therapies. Here we presented for the first time the use of the DNA combing assay in canine cells.

The analysis was performed in two of the canine lymphoma/leukemia cell lines, CLBL-1 and GL-1. The CLBL-1 cell line presented a high basal level of Rad51, and the GL-1 cell line showed the highest expression of Rad51 after etoposide treatment. Both situations may indicate cell replication stress (Figure 2A; Supplementary Figure S2). The replication fork speed in human cells is approximately 2–3 Kb/min (85). In our study, the replication fork speed in the canine cells seemed to be lower, around 1.5 Kb/min for the GL-1 cell line, and 0.86 Kb/min for the CLBL-1 cell line (Figure 6; Supplementary Table S1). It can be concluded that GL-1 cells have a higher replication speed than CLBL-1, which is interesting as the GL-1 cell line’s doubling time is 27.3 h, and for the CLBL-1 it is 19 h (30, 86). The mean values of fork asymmetry were higher than 1 in both cell lines (Supplementary Table S1), indicating a significant number of replication forks terminate asymmetrically in both cell lines (87).

Advanced and novel biomolecular techniques need to be applied in veterinary science in order to improve the quality of the research and stimulate progress in therapy and clinical discoveries. Basic research analysis studying the role of proteins and cellular responses to different treatments is a first step needed to generate a new therapy to treat cancer or any other disease. The structural and functional properties of the principal components of the DDR system are conserved in mammals, but little is known about them specifically in dogs (88, 89). This cellular pathway is under intense investigation in human medicine due to its effects on the clinical aspects of cancer and its potential use in new therapies (90–93). Even where there are effective therapies based on targeting DDR proteins, such as PARP inhibitors (94–96), further investigation is needed due to the fact that cancer cells develop resistance (97). The knowledge about the functioning of the proteins involved in tumor-related pathways, and specifically their behavior in cancer cells is fundamental to finding new targets to be used in therapies.

### 4.4. Importance of validation of techniques and reagents to improve veterinary medicine research

Comparative clinical trials showed analogous results in human and canine patients treated with iniparib and F14512 (topoisomerase II inhibitor), highlighting the similarity of both species in the way naturally occurring cancer and lymphoma respond to therapies (98, 99). Cell lines represent a predictive tool for developing therapies in both human and veterinary medicine (100–102), which means that canine cancer cells represent a tractable model to study cancer that can generate valuable information also for human medicine. We have presented here a set of techniques and reagents validated in selected canine lymphoma/leukemia cell lines, which will facilitate further research in this field. All the data obtained in this work make the selected canine cell lines attractive models to study molecular aspects of lymphoma and leukemia. Experiments on combinations of the tested drugs with inhibitors of the principal components of the studied pathways are planned in the near future.

## 5. Conclusion

To conclude, we propose the use of canine lymphoma/leukemia cells as a model to study DDR in cancer, with ATR, Claspin, Chk1, and Rad51 as promising targets for further analysis. Our results will facilitate further investigation on DDR in canine cancer by identifying validated antibodies for ATR, Claspin, Chk1, p-Chk1, and Rad51, and primers for ATR and Chk1, and bringing numerous opportunities to develop new targeted-anticancer therapies which later may be also implemented in human medicine.

## Data availability statement

The datasets presented in this study can be found in online repositories. The names of the repository/repositories and accession number(s) can be found in the article/Supplementary material.

## Author contributions

AP: conceptualization and project administration. BH-S and AP: methodology. BH-S: software, validation, formal analysis, data curation, writing–original draft preparation, visualization, and funding acquisition. BH-S, AP, PK, and ED: investigation. BO-M: resources. AP and DG: writing–review and editing. AP, DG, and BO-M: supervision. All authors have read and agreed to the published version of the manuscript.

## Funding

The publication was financed by the project “UPWR 2.0:international and interdisciplinary program of development of Wrocław University of Environmental and Life Sciences,” co-financed by the European Social Fund under the Operational Program Knowledge Education Development, under contract No. POWR.03.05.00-00-Z062/18 of June 4, 2019 and by the Polish National Agency for Academic Exchange under Grant No. PPI/APM/2019/1/00044/U/00001. DG was an Agustín de Betancourt Investigador of the Universidad de La Laguna.

## Conflict of interest

The authors declare that the research was conducted in the absence of any commercial or financial relationships that could be construed as a potential conflict of interest.

## Publisher’s note

All claims expressed in this article are solely those of the authors and do not necessarily represent those of their affiliated organizations, or those of the publisher, the editors and the reviewers. Any product that may be evaluated in this article, or claim that may be made by its manufacturer, is not guaranteed or endorsed by the publisher.
