# Supplementary material for: Studying the DNA damage response pathway in hematopoietic canine cancer cell lines, a necessary step for finding targets to generate new therapies to treat cancer in dogs
Source: Front Vet Sci. 2023 Aug 16;10:1227683. doi: 10.3389/fvets.2023.1227683 (PMC10467447; doi:10.3389/fvets.2023.1227683)
Supplement: Supplementary file 2 [file Data_Sheet_2.PDF]

Supplementary Material

Studying the DNA Damage Response pathway in hematopoietic canine cancer cell lines – a necessary step for finding targets to generate new therapies to treat cancer in dogs

Beatriz Hernández-Suárez*, David A Gillespie, Ewa Dejnaka, Piotr Kupczyk, Bożena Obmińska-Mrukowicz, Aleksandra Pawlak1

*** Correspondence:** Corresponding Author: beatriz.hernandez-suarez@upwr.edu.pl

1 Supplementary Data

Protein alignments of the whole length of the protein and the specific epitopes regions were analysed by BLAST. Alignments can be found in a zip file under name SupplementaryMaterial_Alignments.

The RNA sequencing data is available in the repository at Mendeley data: Hernández Suárez, Beatriz; Pawlak, Aleksandra (2023), "RNA sequencing CLBL-1 and GL-1 cell lines", Mendeley Data, V1, doi: 10.17632/5hg29rjtdc.1.

2 Supplementary Figures and Tables

2.1 Supplementary Figures

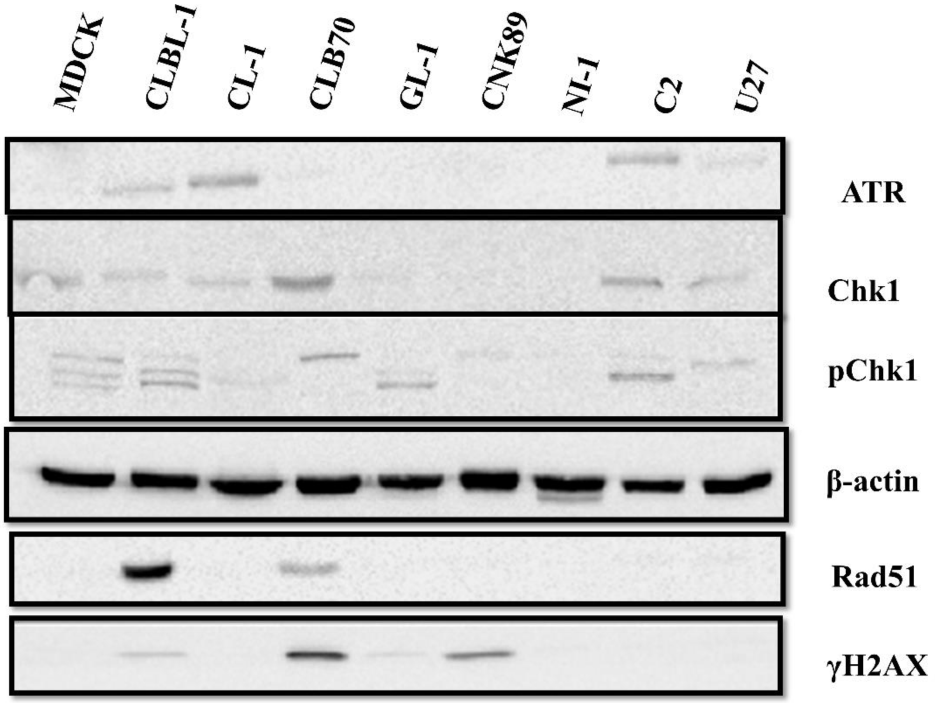

Supplementary Figure 1 (S1). First screening looking for basal DDR protein expression levels in the different canine cancer cell lines. The different panel of cell used were MDCK (Madin-Darby canine kidney), CLBL-1 (B-cell lymphoma), CL-1 (primitive αβ T-cell leukemia), CLB70 (B-cell chronic lymphocytic leukemia), GL-1 (B-cell leukemia) and CNK-89 (NK-cell lymphoma), NI-1 (canine mastocytoma cell line), C2 (canine mastocytoma cell line), CMT-U27 (canine mammary cancer cell line) (1–4).

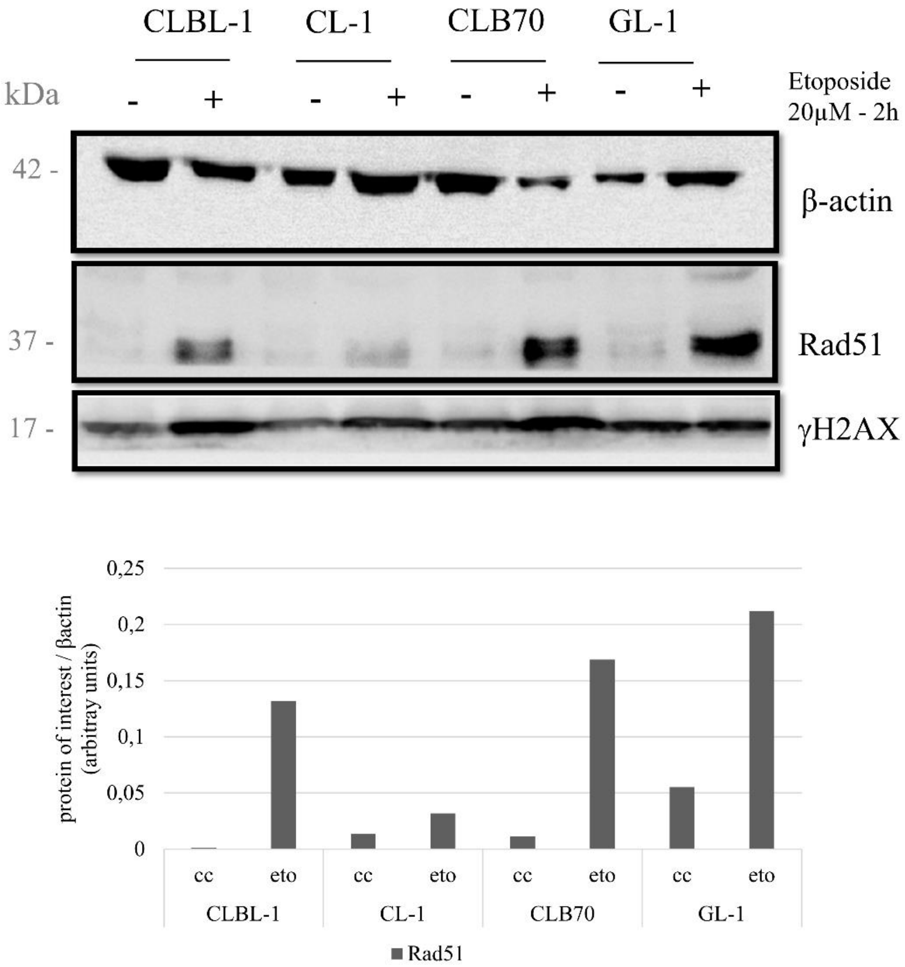

Supplementary Figure 2 (S2). Expression of Rad51 protein before and after etoposide treatment in canine lymphoma/leukemia cell lines. Expression of Rad51protein increased greatly after treatment with etoposide. Quantification compares the expression level of the protein of interest to the expression level of the loading control, β-actin. Representative data from a single experiment.

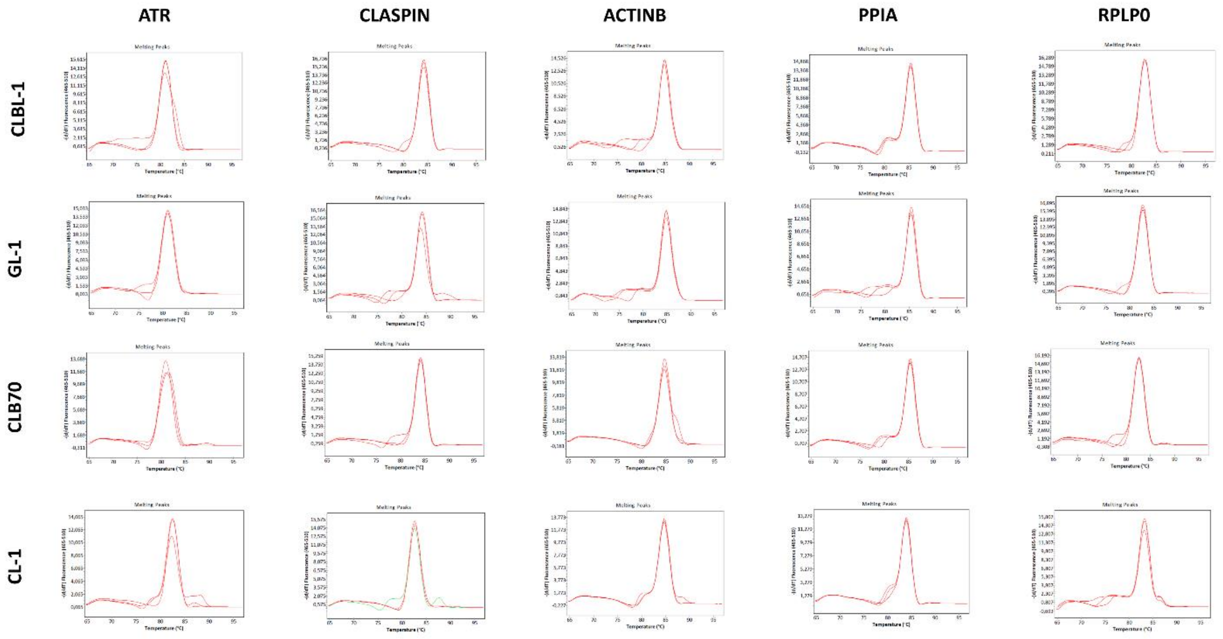

Supplementary Figure 3 (S3). Melting curves of the products obtained after the qPCR analysis. A single peak is observed for all genes, indicating a single amplicon and validating the specificity of the primers.

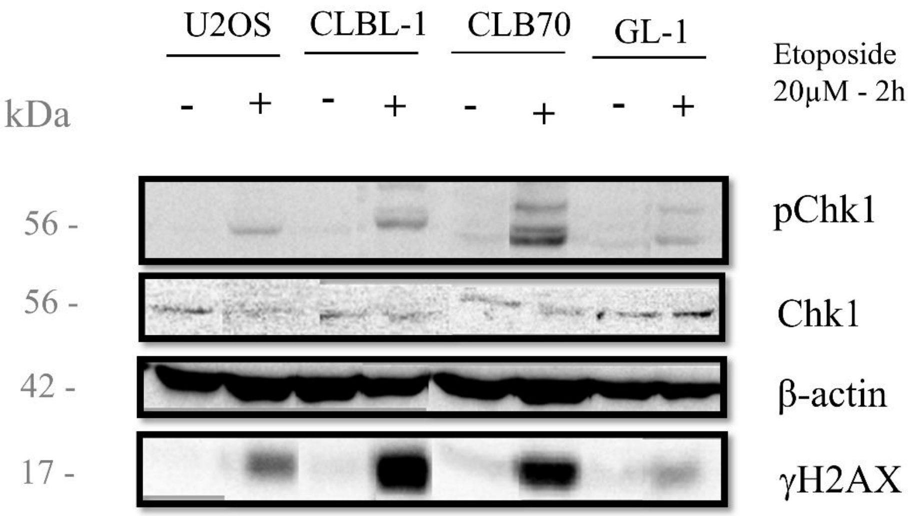

Supplementary Figure 4 (S4). The levels of phosphorylation of Chk1 and gH2AX are increased after the treatment with the DNA damaging agent etoposide at a concentration of 20uM for 2 hours in multiple canine lymphoma/leukemia cancer cells (CLBL-1, CLB70, and GL-1) and in the control human cell line U2OS (human osteosarcoma cell line).

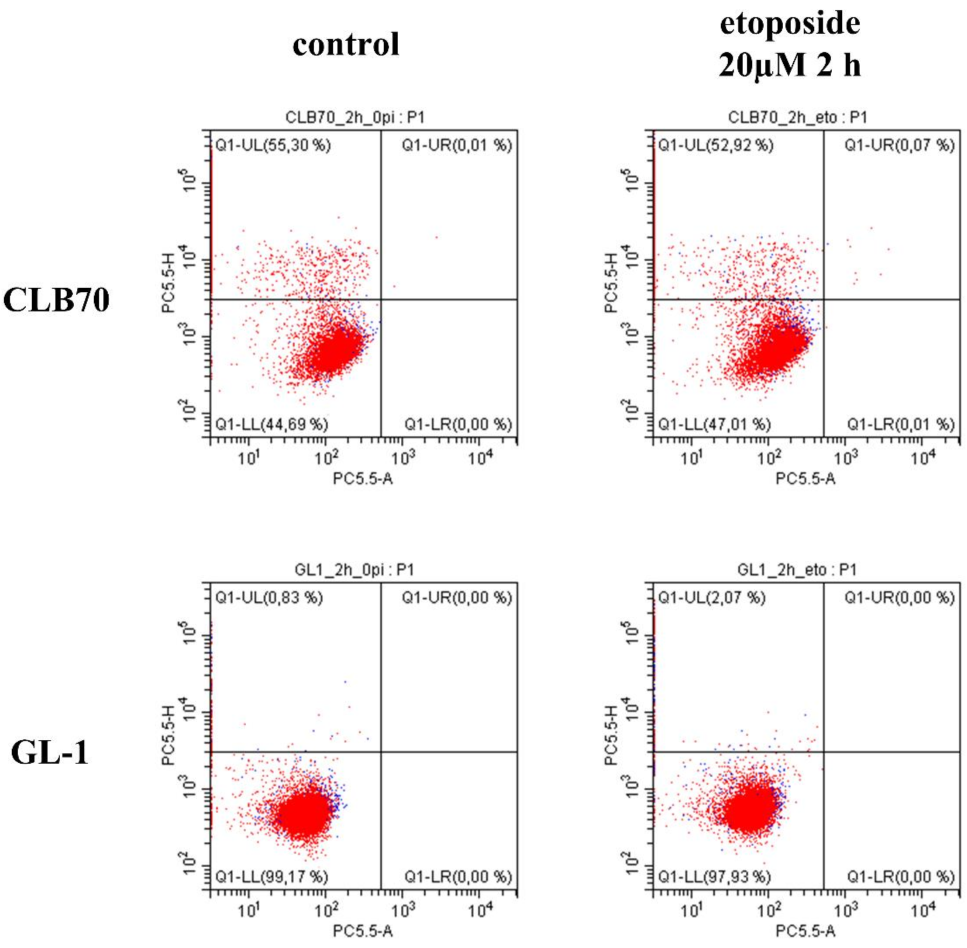

Supplementary Figure 5 (S5). Flow cytometry analysis have shown that no cell dead is observed after treatment with etoposide 20µM for 2 hours. 2 million of cells of GL-1 and CLB70 cell lines well culture per condition in a 25 cm2 flask in 5 mL of media. The cells were suspended in a PBS with PI (PI concentration, 1 µg/mL) for 10 minutes at room temperature and later proceed in a CytoFlex cytometer and analysed using the CytoExpert software.

2.2 Supplementary Tables

Supplementary Table 1 (S1). Descriptive data.

**Variable**	**Cell line=GL-1 Descriptive Statistics (Spreadsheet en gl1 clbl1 stat)**				
	Valid N	Mean	Minimum	Maximum	Std.Dev.
**FV**	193	1,523946	0,269067	5,318267	0,872562

**Variable**	Cell line=CLBL-1 Descriptive Statistics (Spreadsheet en gl1 clbl1 stat)				
	Valid N	Mean	Minimum	Maximum	Std.Dev.
**FV**	168	0,858464	0,193600	3,813200	0,441150

**Variable**	**Cell line=CLBL-1 Descriptive Statistics (gl1 clbl1 stat)**				
	Valid N	Mean	Minimum	Maximum	Std.Dev.
**FA**	10	1,270589	1,000000	1,833162	0,285005

**Variable**	Cell line=GL-1 Descriptive Statistics (gl1 clbl1 stat)				
	Valid N	Mean	Minimum	Maximum	Std.Dev.
**FA**	19	1,326321	1,000000	2,981635	0,445235

References

1. Irvine JD, Takahashi L, Lockhart K, Cheong J, Tolan JW, Selick HE, et al. MDCK (Madin-Darby Canine Kidney) Cells: A Tool for Membrane Permeability Screening. J Pharm Sci. 1999 Jan;88(1):28–33.

2. Hadzijusufovic E, Peter B, Herrmann H, Rülicke T, Cerny-Reiterer S, Schuch K, et al. NI-1: a novel canine mastocytoma model for studying drug resistance and IgER-dependent mast cell activation. Allergy. 2012 Jul 15;67(7):858–68.

3. DeVinney R, Gold WM. Establishment of Two Dog Mastocytoma Cell Lines in Continuous Culture. Am J Respir Cell Mol Biol. 1990 Nov;3(5):413–20.

4. Hellmén E. Characterization of four in vitro established canine mammary carcinoma and one atypical benign mixed tumor cell lines. In Vitro Cell Dev Biol Anim. 1992 May;28(5):309–19.
